# Identifying the Carcinogenic Mechanism of Malignant Struma Ovarii Using Whole-Exome Sequencing and DNA Methylation Analysis

**DOI:** 10.3390/cimb45030118

**Published:** 2023-02-23

**Authors:** Hitomi Yamashita, Kentaro Nakayama, Kosuke Kanno, Tomoka Ishibashi, Masako Ishikawa, Seiya Sato, Koji Iida, Sultana Razia, Satoru Kyo

**Affiliations:** Department of Obstetrics and Gynecology, Shimane University School of Medicine, Izumo 693-8501, Japan

**Keywords:** malignancy, struma ovarii, whole-exome sequencing, DNA methylation

## Abstract

Background: Since malignant struma ovarii is a very rare disease, its carcinogenic mechanism has not been elucidated. Here, we sought to identify the genetic lesions that may have led to the carcinogenesis of a rare case of malignant struma ovarii (follicular carcinoma) with peritoneal dissemination. Methods: DNA was extracted from the paraffin-embedded sections of normal uterine tissues and malignant struma ovarii for genetic analysis. Whole-exome sequencing and DNA methylation analysis were then performed. Results: Germline variants of *RECQL4*, *CNTNAP2*, and *PRDM2*, which are tumor-suppressor genes, were detected by whole-exome sequencing. Somatic uniparental disomy (UPD) was also observed in these three genes. Additionally, the methylation of *FRMD6-AS2*, *SESN3*, *CYTL1*, *MIR4429*, *HIF3A*, and *ATP1B2*, which are associated with tumor growth suppression, was detected by DNA methylation analysis. Conclusions: Somatic UPD and DNA methylation in tumor suppressor genes may be associated with the pathogenesis of malignant struma ovarii. To our knowledge, this is the first report of whole-exome sequencing and DNA methylation analysis in malignant struma ovarii. Genetic and DNA methylation analysis may help elucidate the mechanism of carcinogenesis in rare diseases and guide treatment decisions.

## 1. Introduction

Struma ovarii is defined as a mature cystic teratoma that contains more than 50% thyroid tissue. Struma ovarii accounts for approximately 5% of mature cystic teratomas [1]. The malignant transformation of struma ovarii occurs in only 5–10% of struma ovarii cases. Additionally, the frequency of distant metastases is very low, occurring in only 5–6% of cases [2,3]. Given its rarity, malignant struma ovarii’s carcinogenic mechanism has not been elucidated and no standard treatment has been established. Surgery and ^131^I therapy have been reported as the main treatment for malignant struma ovarii [4]. However, there have been few reports on the treatment of residual tumors after surgery and ^131^I therapy. Here, we report a case of malignant struma ovarii with peritoneal dissemination. The patient was treated with surgery, ^131^I therapy, and chemotherapy. However, the peritoneal dissemination gradually increased. Therefore, genetic analysis was performed to elucidate the cause of malignant struma ovarii and provide the guidance for treatment.

## 2. Materials and Methods

This study was conducted according to the ethical standards of national and international guidelines as well as the Declaration of Helsinki and was approved by the institutional review board (Shimane University Hospital). Tumor specimens were collected after obtaining written consent from the patient with the approval of the Facility Ethical Committee (Shimane University Hospital; approval no. 2004-0381).

Genetic analysis was performed using the PleSSision exome^®^ test (MITSUBISHI SPACE SOFTWARE, Tokyo, Japan) [5]. Hypermutated and ultra-hypermutated statuses were defined as ≥10 and ≥100 SNVs/Mbp, respectively, as previously described [6]. For genetic analysis, DNA was extracted from the paraffin-embedded sections of normal uterine tissues and malignant struma ovarii. The annotation and curation of the sequencing data were performed using the bioinformatics pipeline GenomeJack (MITSUBISHI SPACE SOFTWARE).

We used the Infinium MethylationEPIC BeadChip^®^ (Illumine, San Diego, CA, USA) to analyze the methylation rates of normal uterine tissues and malignant struma ovarii. DNA methylation levels were expressed as β values in normal tissues and malignant struma ovarii. The β value ranges from 0 to 1, and approaches 1 as the methylation rate increases. The difference in methylation rate between normal tissues and malignant struma ovarii for the genes was obtained by the log ratio. The higher the methylation rate in malignant struma ovarii compared to that in the normal tissue, the higher the log ratio. We extracted the top 50 genes with large differences in methylation rates and picked up genes that may have been involved in tumor growth due to methylation changes in malignant struma ovarii.

## 3. Results

A 29-year-old unmarried, nulliparous woman visited a gynecological clinic due to irregular vaginal bleeding. The patient was referred to our hospital for further examination. She had undergone a laparoscopic left ovarian cystectomy 4 years prior to the visit to our hospital because of a mature cystic teratoma including thyroid tissue.

**Figure 1:**
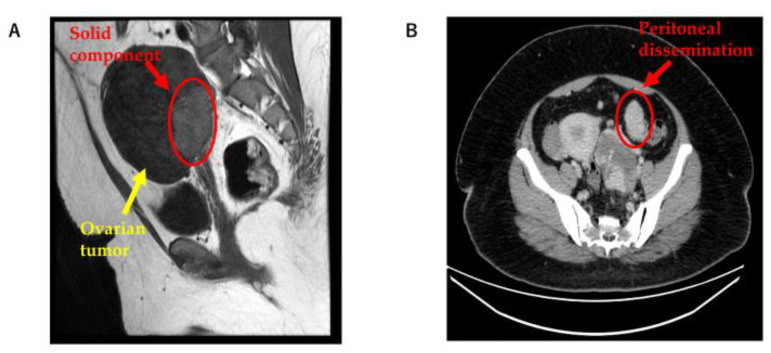
(**A**) MRI showing a solid component in the left ovarian tumor; and (**B**) CT showing peritoneal dissemination. MRI: magnetic resonance imaging; CT: computed tomography.

A multiple cystic mass was detected in the left ovary by transvaginal ultrasonography. To further investigate the left ovarian tumor, magnetic resonance imaging (MRI) and computed tomography (CT) were performed. MRI showed a solid component in the left ovarian tumor, and CT showed peritoneal dissemination (Figure 1A,B). Ovarian cancer was suspected based on the medical examination and imaging findings. Therefore, open surgery was selected as the treatment.

**Figure 2:**
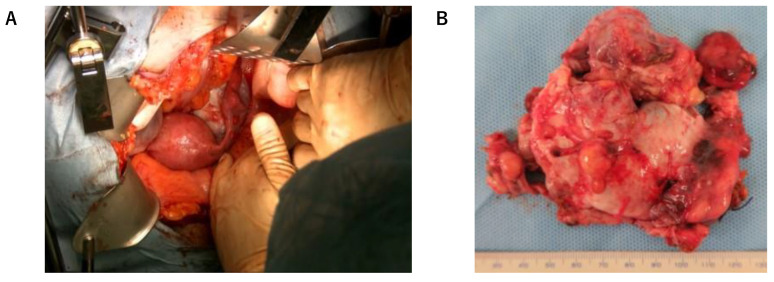
(**A**) Left ovarian tumor at laparotomy; (**B**) Resected tumor.

Intraoperatively, the left ovarian tumor was approximately 7 cm in size and adhered to the rectum. Extensive peritoneal dissemination (max. 3 cm) was observed (Figure 2A,B). First, we removed the left ovary. The intraoperative pathological diagnosis of the tumor in the left ovary was malignant struma ovarii (follicular carcinoma). Since the patient elected to not preserve fertility, total hysterectomy, bilateral salpingo-oophorectomy, and omentectomy were performed. After the operation, >2 cm of peritoneal dissemination remained (suboptimal surgery).

**Figure 3:**
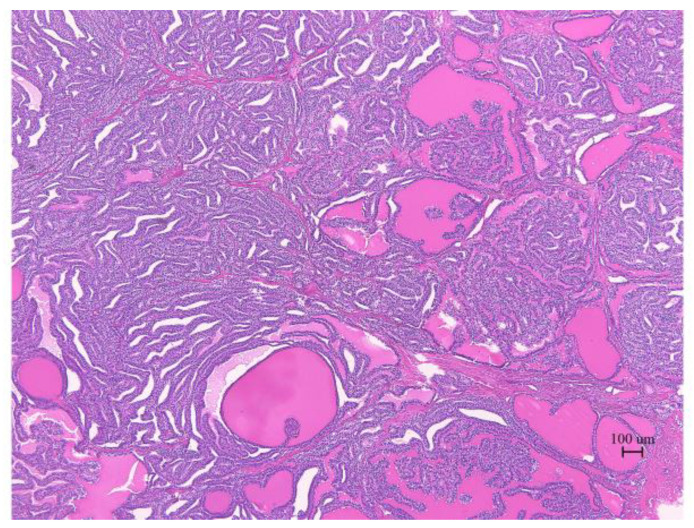
Micrograph (H&E staining) of the malignant struma ovarii. The thyroid follicular structure was the main component.

The pathological analysis of the resected tumor indicated that the histological type was a highly differentiated follicular carcinoma (Figure 3). No findings of papillary carcinoma, such as psammoma body, intranuclear cytoplasmic inclusion, ground glass nuclei, or nuclear groove, were observed. Similar histology was observed on the posterior wall of the uterus, omentum, and mesentery. This case was diagnosed as Stage IIIC by the 2014 International Federation of Gynecology and Obstetrics (FIGO).

Approximately 8% of patients with malignant struma ovarii are reported to have hyperthyroidism [7], but the patient was euthyroid (thyroid-stimulating hormone, 0.7 µU/mL; free T3, 2.6 pg/mL; free T4, 1.4 ng/dL). Because the ovarian metastasis of thyroid cancer must be ruled out for the diagnosis of malignant struma ovarii, we examined the removed thyroid gland but found no malignant findings. After thyroidectomy, ^131^I therapy (100 mCi) was used to treat the residual tumor. After ^131^I therapy, the peritoneal dissemination gradually increased. Therefore, chemotherapy (paclitaxel, carboplatin, and bevacizumab) was administered based on the treatment for the residual ovary tumor. However, the peritoneal dissemination increased slowly during chemotherapy.

Most cases of malignant struma ovarii are only treated with surgery and ^131^I therapy, and few case reports have shown other types of treatment. Therefore, we performed the genetic and DNA methylation analyses to the identify genetic lesions that may have led to the carcinogenesis of malignant struma ovarii that was intractable even after surgery and ^131^I therapy.

**Figure 4:**
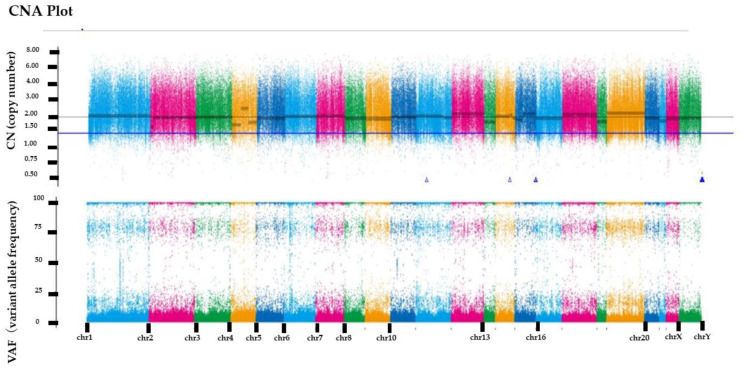
Copy number alteration plot (CNA plot). The horizontal axis represents the position of the chromosome and the vertical axis represents the copy number of the genes.

**Figure 5:**
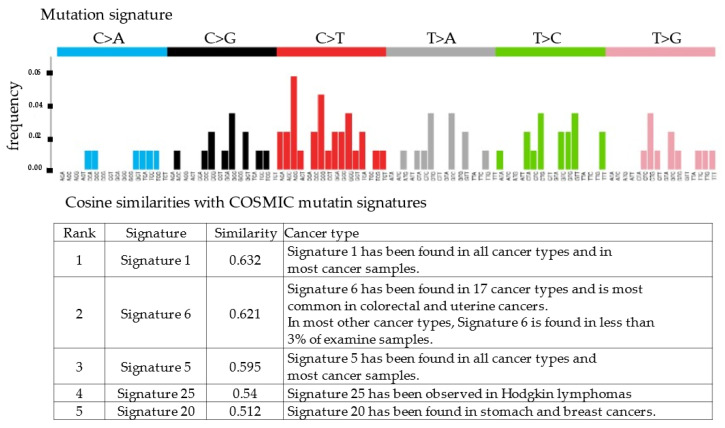
Cosine similarities with COSMIC mutation signatures. Signature 1 showed the highest similarities with the mutation profile of the patient.

*RecQ like heliccase4* (*RECQL4*), *CNTNAP2*, and *PRDM2* variants, which are tumor suppressor genes, were detected by whole-exome sequencing. All three variants were germline variants and revealed a somatic uniparental disomy (somatic UPD). Variant positions and variant allele frequencies are shown in Table 1. However, no actionable variants and no copy number alterations were identified in the known oncogenes. The copy number plot was normal and therefore, the tumor was considered homologous recombination proficient (HRP) (Figure 4). Moreover, it was determined to be microsatellite-stable given that the microsatellite instability was 3.82%. The tumor mutation rate was 1.73 SNVs/Mbp, indicating no tumor mutation burden. We also examined the mutation signatures of the malignant struma ovarii. The most prevalent mutational signature was signature 1, which has been found in all cancer types and in most cancer samples (Figure 5).

The DNA methylation analysis detected the methylation of *FRMD6-AS2*, *SESN3*, *CYTL1*, *MIR4429*, *HIF3A*, and *ATP1B2* in malignant struma ovarii (Table 2). All these genes were reportedly associated with tumor growth suppression; thus, it is possible that the methylation of these genes was involved in the carcinogenesis of malignant struma ovarii [8,9,10,11,12,13,14].

## 4. Discussion

Malignant struma ovarii is a very rare disease, with only approximately 200 case reports in the published literature. Only 8% of patients with malignant struma ovarii have hyperthyroidism; thus, malignant struma ovarii is rarely diagnosed based on the symptoms of hyperthyroidism [7]. In addition, preoperative diagnosis is very difficult because the MRI findings of malignant struma ovarii are nonspecific. Therefore, malignant struma ovarii is usually diagnosed after surgery. Since malignant struma ovarii is a rare disease, its carcinogenic mechanism is unknown, and no standard treatment has been established. In case reports, surgery, thyroidectomy, and ^131^I therapy are the most common treatments [4]. Here, we report a rare case of malignant struma ovarii with peritoneal dissemination. The patient was treated with surgery, ^131^I therapy, and chemotherapy. However, peritoneal dissemination gradually increased. Therefore, we sought to identify the genetic lesions that may have led to the development of malignant struma ovarii, which can provide guidance for treatment. To that aim, we performed genetic and DNA methylation analyses. We detected the germline variants of *RECQL4*, *CNTNAP2*, and *PRDM2*, which are tumor suppressor genes. Furthermore, somatic UPD was observed in these three genes. In addition, the methylation of *FRMD6-AS2*, *SESN3*, *CYTL1*, *MIR4429*, *HIF3A*, and *ATP1B2*, which are associated with tumor growth suppression, was also detected. These findings suggest that somatic UPD and DNA methylation in tumor suppressor genes may be associated with the carcinogenesis of malignant struma ovarii.

Papillary carcinoma is the most common histological subtype of malignant struma ovarii, and follicular carcinoma, as seen in the present case, is rare [15]. Total thyroidectomy is recommended to confirm the diagnosis of malignant struma ovarii by excluding ovarian metastases from primary thyroid cancer to improve the effectiveness of ^131^I therapy by allowing preferential uptake into residual struma or metastasis [16].

Previous reports have described different surgical procedures for malignant struma ovarii. In a population-level analysis of 68 patients, 33.0% of patients underwent unilateral oophorectomy, 28.6% underwent bilateral oophorectomy, 28.6% underwent oophorectomy and omentectomy, and 4.8% underwent debulking surgery [4]. The average age of the 68 patients was 43 years, and the overall survival rates at 5 and 10 years were 96.7% and 94.3%, respectively [4]. The 5-year and 10-year recurrent rates of the patients with MSO confined to the ovary were 27.1% and 35.2%, respectively [17]. Although the survival rate of malignant struma ovarii is relatively good, it is unclear whether conservative surgery is possible in young patients with peritoneal dissemination, as in the present case. The large tumor size (≥10 cm), adhesion to the surrounding organs, ascites (≥1 L), capsule rupture, stromal component higher than 80%, extensive papillary carcinoma, especially with solid components, necrosis, and more than 5 mitoses per 10 high-power fields have been reported as factors that predict malignancy [18]. Follicular cancer may be more susceptible to hematogenous metastasis than papillary cancer, as it is observed in primary thyroid carcinoma [19,20]. If more cases of malignant struma ovarii are accumulated, the prognosis can be predicted better, and treatment guidelines may be determined based on the prognosis prediction. Conservative surgery may be acceptable in cases without poor prognostic factors.

Very few studies have reported the treatment of malignant struma ovarii by means other than surgery and ^131^I therapy. However, one case report described a patient with lung and bone metastases who survived more than 20 years after treatment with tegafur-uracil, paclitaxel/carboplatin, and oral etoposide [19]. In malignant tumors for which standard treatment has not been established or that are resistant to conventional treatments, genetic analysis using next-generation sequencing is now being used to guide treatment decision making. Associations between histological types and genetic variants were reported in primary thyroid carcinoma. *BRAF^V600E^, RAS*, and rearranged during transfection/papillary thyroid carcinoma (*RET/PTC*) variants are common in primary papillary thyroid carcinomas [21,22]. In primary PTCs, *RET/PTC* rearrangements correlate with young age, tumor morphology, and a high frequency of lymph node metastases [23]. *BRAF* variants have been reported to be associated with aging, advanced disease, classical papillary histology, and poor prognosis [24,25]. Conversely, in primary follicular thyroid carcinomas, *RAS* variants and *PAX8*/Peroxisome Proliferator-Activated receptor γ (PPAR**γ**) fusions are found in approximately 80% of cases [26].

Several genetic variants have been reported in malignant struma ovarii. *RET/PTC1* and *RET/PTC3* rearrangements have been observed in follicular variant PTC originating from struma ovarii [27]. *BRAF^V600E^*, *KRAS* variants, *NRAS* variants, and *HRAS* variants have also been observed in papillary carcinoma originating from struma ovarii [28,29,30,31,32,33]. Primary PTC and malignant struma ovarii with papillary carcinoma have similar genetic variants and may share a mechanism of carcinogenesis [31]. *BRAF* variants in primary PTC were reported to overexpress cytotoxic T-lymphocyte-associated protein 4 (CTLA4) and programmed cell death ligand-1 (PD-L1), which are responsible for immunosuppression [34,35]. These data may provide the rationale for immunotherapy in PTC originating from struma ovarii. However, the follicular carcinomas of malignant struma ovarii are rarer than papillary carcinomas; thus, there are only a few reports of the genetic analyses of these tumors. No major oncogenic gene variants, including *KRAS, BRAF, P53,* or *PAX8/*PPAR**γ** gene fusions, have been found in the genetic analysis of follicular carcinoma originating from struma ovarii [36]. These cases suggest that the mechanism of carcinogenesis differs between the primary follicular thyroid carcinoma and follicular carcinoma originating from malignant struma ovarii [36]. To our knowledge, this is the first report of whole-exome sequencing and DNA methylation analysis in malignant struma ovarii with follicular carcinoma. *RECQL4*, *CNTNAP2*, and *PR/SET domain 2 (PRDM2)* variants (somatic UPD) were detected by whole-exome sequencing. RECQL4 is a DNA helicase that belongs to the RecQ helicase family. DNA helicases unwind double-stranded DNA into single-stranded DNA and regulate chromosome segregation. Therefore, *RECQL4* is deeply involved in maintaining genome stability and is classified as a tumor suppressor gene [37]. *CNTNAP2* encodes a member of the neurexin family that functions as a cell adhesion molecule and receptor. *CNTNAP2* was reported to be an independent prognostic factor, with a high CNTNAP2 mRNA expression level associated with a good prognosis in glioma. Therefore, *CNTNAP2* is also considered a tumor suppressor gene [38]. *PRDM2* is a member of a nuclear histone/protein methyltransferase superfamily and a tumor suppressor gene. *PRDM2* encodes a zinc finger protein that can bind to retinoblastoma protein, estrogen receptor. PRDM2 is involved in tumor suppression, and its expression is often silenced in many cancer types [39]. In this study, these three tumor suppressor genes exhibited somatic UPD, suggesting that they may be deeply involved in the development of malignant struma ovarii. DNA methylation analysis also revealed the methylation of multiple genes (*FRMD6-AS2*, *SESN3*, *CYTL1*, *MIR4429*, *HIF3A*, and *ATP1B2*) associated with tumor growth suppression. The methylation of these genes leads to tumor growth because DNA methylation inhibits transcription. Therefore, we believe that the methylation of these genes is related to the development of malignant struma ovarii.

Various analyses, such as transcriptome, methylome profiling, digital histopathology, and multiplexed immunofluorescence have been performed in high-grade serous ovarian carcinoma. The characteristics of high-grade serous carcinoma tumors have gradually become clear, leading to the discovery of new therapeutic strategies [40,41,42]. However, since malignant struma ovarii is a rare tumor, its carcinogenic mechanism remains unknown and no effective treatment has been established. Genetic and epigenomic analyses, such as those conducted in this study, will provide clues to clarify the carcinogenic mechanism of malignant struma ovarii.

## 5. Conclusions

This study is the first report of whole-exome sequencing and DNA methylation analysis in malignant struma ovarii. Genetic and DNA methylation analysis may help elucidate the mechanism of carcinogenesis in rare diseases and guide treatment decisions.

## Figures and Tables

**Table 1:** Actionable variants detected by whole-exome sequencing.

Actionable Variants	Variant Allele Frequency (%)	Copy Number
g*RECQL4* p.E976K	82.4	1.9
g*CNTNAP2* p.I172T	79.6	2.1
g*PRDM2* p.A935S	77.4	2.1

**Table 2:** The DNA methylation analysis detected the methylation of *FRMD6-AS2*, *SESN3*, *CYTL1*, *MIR4429*, *HIF3A*, and *ATP1B2* in malignant struma ovarii.

Gene Symbol	Type of Gene	RefSeq_Accession	Log Ratio	Average β Value of Malignant Struma Ovarii	Average β Value of Normal Uterine Tissues
*FRMD6-AS2*	ncRNA	NR_051990	2.641554295	0.848721886	0.136012343
*SESN3*	protein-coding	NM_001271594	3.652856573	0.705986834	0.056127694
*CYTL1*	protein-coding	NM_018659	3.427866379	0.70975	0.06595
*MIR4429*	ncRNA	NR_039627	2.562496834	0.751119105	0.127151181
*HIF3A*	protein-coding	NM_152796	3.017858731	0.708329124	0.08745187
*ATP1B2*	protein-coding	NM_001303263	1.959788684	0.819775968	0.210736617

## Data Availability

Data from the current study are available from corresponding author (K.N.).

## Additional Information

Correspondence and requests for materials should be addressed to K.N.
